# White blood cell and neutrophil trends in likely early-onset neonatal sepsis: a sex-stratified analysis before and after antibiotic therapy

**DOI:** 10.3389/fped.2026.1917831

**Published:** 2026-10-02

**Authors:** Isaline Eggermont, Andrea Nebbioso, Alfredo Vicinanza, Phu-Quoc Lê, Nancy Vitali, Gwenaëlle Augé, Nicolas Lefèvre

**Affiliations:** 1 Université Libre de Bruxelles (ULB), Hôpital Universitaire de Bruxelles (H.U.B), Hôpital Universitaire des Enfants Reine Fabiola (HUDERF), Brussels, Belgium; 2 Department of Pediatrics, Hôpitaux Iris Sud (HIS), Brussels, Belgium

**Keywords:** absolute neutrophil count, antibiotic treatment, early-onset neonatal sepsis, kinetics, sex differences, white blood cell

## Abstract

**Introduction:**

Early-onset neonatal sepsis (EONS) requires rapid diagnosis, yet the interpretation of white blood cell (WBC) and absolute neutrophil count (ANC) is complicated by physiological postnatal changes. While sex differences in neutrophil responses are known in older populations, limited data exist in neonates. This study aimed to investigate the kinetics of WBC and ANC in neonates with likely EONS and evaluate potential sex differences, including responses to antibiotic initiation.

**Methods:**

We reviewed neonates treated at our center with antibiotics for likely EONS (suspected EONS with elevated CRP) between 2017 and 2022. WBC and ANC values were collected at standardized intervals before and after antibiotic initiation. Data were analyzed using piecewise linear mixed-effects regression models stratified by sex and timing of antibiotic initiation.

**Results:**

Of the 506 neonates included, 304 (60.1%) were males and 202 (39.9%) females. Female neonates had higher ANC at birth (10,366 *vs.* 9,573 cells/*μ*L, *p* = 0.012) and after antibiotic initiation (14,519 *vs.* 13,359 cells/μL, *p* = 0.042). During the first 12 h of life, ANC increased in both sexes (673 cells/μL/hour in females, 603 cells/μL/hour in males, *p* = 0.001), with no difference in rate. Among neonates treated at birth, WBC counts increased more rapidly in females (557 *vs.* 380 cells/μL/hour, *p* = 0.020). In contrast, neonates treated after birth showed a similar decline in WBC and ANC regardless of sex.

**Conclusion:**

Female neonates with likely EONS consistently demonstrated higher ANC values and a more pronounced WBC increase in the early hours of life and after antibiotic initiation. This may reflect sex differences, potentially due to X-linked genes involved in the innate immune response. These findings support sex-specific interpretation of inflammatory markers in the evaluation of likely EONS evaluation and warrant further investigation into underlying immune differences.

## Introduction

Early-onset neonatal sepsis (EONS) typically occurs within the first 72 h of life (1) and remains a critical concern in neonatal intensive care units, necessitating prompt identification and intervention. Most often, a newborn will present with non-specific signs and symptoms suggestive of EONS without a causative agent being isolated in sterile fluids (e.g., blood, cerebrospinal fluid or pleural fluid). This particular presentation is referred to as clinical sepsis (2). White blood cell (WBC) and absolute neutrophil counts (ANC) are widely used biomarkers when screening symptomatic and asymptomatic newborns at risk for neonatal sepsis (NS) (3). However, interpreting these parameters in neonates is complex due to their dynamic postnatal changes and the influence of various factors such as gestational age or type of delivery (3–5). In the initial hours of life, neonates exhibit a physiological transient leukocytosis, with WBC counts peaking around 6 to 8 h after birth. Neutrophil counts follow a similar trajectory, with ANC reaching their peak during the first 12 h (5–8). Several studies found that in the first hour of life, only low WBC counts and ANC are associated with an increased risk of EONS (6, 8, 9). Indeed, sepsis is one of the few conditions that can cause a decrease in ANC in neonates, with the lower standard limit for ANC at birth being 3,500/µL in term infants (4, 7, 9). The initiation of antibiotic therapy in likely EONS cases further complicates the interpretation of WBC and ANC. Understanding how antibiotic administration influences WBC and ANC trajectories is crucial for refining diagnostic and therapeutic strategies in neonates with EONS.

Compared with children, adults tend to have more stable WBC and ANC, yet notable sex differences persist. Females typically exhibit higher ANC than males, a disparity attributed mainly to hormonal influences—particularly estradiol and progesterone—which prolong neutrophil survival and enhance activation (10, 11). In contrast, male neutrophils often display a less mature and less activated phenotype, exhibiting distinct metabolic profiles (10, 11). To disentangle the role of sex hormones from X chromosome gene dosage, Gupta et al. analyzed ANC from healthy prepubescent males and females. They found no significant sex-based differences in interferon-stimulated gene expression, unlike in their cohort of young adults, suggesting a hormonal influence (10). However, in prepubescent girls with *in vivo* acute inflammation such as pneumonia, pyelonephritis or bronchiolitis, and with chronic inflammation such as asthma and cystic fibrosis, Casimir et al. found a higher ANC, suggesting a difference associated with the X chromosome (12).

Given the distinct hormonal environment of the neonatal period compared with that of prepubertal children and adults, we aimed to characterize WBC and ANC kinetics during early neonatal life in neonates with suspected EONS and to assess whether these trajectories vary by sex and antimicrobial treatment.

## Methods

### Operational definitions

Early-onset neonatal sepsis (EONS): NS occurring within the first 72 h of life (1).

Clinical symptoms: Fever (> 38°), hypothermia (< 36,0°), poor sucking, haemodynamic instability, apnoea, respiratory distress, cyanosis, grunting, irritability, recurrent hypoglycaemia, and purpura-like cutaneous rash.

Infectious risk factors: maternal fever, prolonged membrane rupture, maternal group B streptococcus colonization, chorioamnionitis, maternal antibiotic treatment and integrated calculation of risk using the neonatal sepsis risk calculator (13).

Abnormal laboratory findings: CRP > 20 mg/L or WBC < 4,000/μL.

Suspected EONS: neonates treated with antibiotics because the clinician suspected EONS based on a combination of at least two of the following three criteria:

1. Presence of at least one clinical symptom.
2. Presence of at least one infectious risk factor.
3. Presence of at least one abnormal laboratory finding.

Unlikely EONS: suspected EONS (as previously defined) with CRP measurements never higher than 10 mg/L.

Likely EONS: suspected EONS (as previously defined) with at least one CRP measurement higher than 10 mg/L.

Confirmed EONS: suspected EONS with positive culture of blood or another sterile site.

Unconfirmed EONS: culture-negative suspected EONS.

### Study design and setting

Our study is an observational monocentric retrospective cohort study conducted in the neonatal unit (NU) and the maternity ward (MW) of Iris South Hospitals (HIS). HIS MW has an average of 2,000 live births per year, and the NU has approximately 400 patients admitted per year with a multi-ethnic patient population. As our NU does not provide neonatal intensive care, preterm neonates before 32 weeks of gestational age and/or neonates with a birth weight under 1,500 grams are transferred to larger centers before birth (*in utero*) or after birth if imminent delivery occurs. Intermediate neonatal care can be provided in HIS NU with availability of incubators, non-invasive ventilation, oxygen therapy, parenteral nutrition via central lines, and nasogastric tube feeding. In our NU and MW, the first-line treatment of NS is a combination of amoxicillin-amikacin (five days of amoxicillin and two days of amikacin), and the second-line treatment is a combination of amoxicillin-cefotaxime. The neonatal sepsis risk calculator has been systematically used to assess the risk of EONS since January 2020. Neonates with suspected EONS in HIS are systematically admitted to NU for the first few hours of treatment. On the other hand, well-appearing neonates presenting infectious risk factors are admitted to MW, where WBC and ANC are measured at 0, 12, and 24 h of life before any antibiotic treatment. If EONS is suspected because of abnormal laboratory findings or development of neonatal symptoms, then the neonate is admitted to NU. In the HIS neonatal unit, blood culture was systematically obtained before antibiotic initiation in all neonates with suspected EONS whenever blood sampling was technically feasible. WBC and ANC were subsequently measured at 12 and 30 h after antibiotic initiation.

### Study population and eligibility criteria

The study population is identical to that described in our article about C-reactive protein (CRP) kinetics and EONS (14). Inclusion criteria were: all neonates born in the facility (HIS) from January 2017 to December 2022 and starting antibiotic treatment within the first 72 h of life. Exclusion criteria were: unlikely EONS, as previously defined in operational definitions. For patients born at our facility and transferred later, only blood samples collected in HIS were included in the analysis.

### Data collection

Patients’ files were retrospectively reviewed to collect the data. The following information was collected: date of birth, type of delivery, gestational age at birth, birth weight and its Z-score, sex, presence of risk factors for NS (meconial liquid, maternal fever, prolonged membrane rupture, maternal group B streptococcus colonization, presence of chorioamnionitis and maternal antibiotic treatment), data of clinical presentation of neonates (fever, jaundice, respiratory distress, timing of antibiotic start and blood culture result), WBC count, ANC and time of sampling.

### Statistical analyses

Frequencies and proportions were calculated for categorical variables. The Fisher's exact test was used to determine the association between categorical variables. Continuous variables were described using median and interquartile range. The Mann–Whitney U test was performed for continuous variables. By convention, time was expressed in hours, and WBC and ANC were reported in cells/µL.

We decided to describe WBC and ANC kinetics according to two different time axes. The first time-axis begins at birth (T_0_) and is used to describe the kinetics of WBC and ANC before the initiation of antibiotic treatment. The second time-axis starts at the initiation of antibiotics (T_0_) and is used to describe the kinetics of WBC and ANC following treatment initiation. We fitted two identical models (one for WBC and one for ANC) for each time axis. To analyze the kinetics of WBC and ANC in male and female neonates, we fitted a piecewise linear mixed-effects regression model with birth as T_0_ and a knot at 12 h of life; observations beyond 24 h of life or after initiation of antibiotic treatment were excluded. Sex was the only covariate in this model. A second piecewise linear mixed-effects regression model was fitted, with the initiation of antibiotic treatment as T_0_ and a knot at 12 h post-initiation – observations before treatment or more than 30 h after initiation were excluded. Sex and the timing of antibiotic initiation (at birth *vs.* after birth) were the two covariates in this model. In all models, we introduced a random effect on intercept and slope, and we used an unstructured covariance matrix to account for serial correlation. A *p*-value of < 0.05 was considered statistically significant. Statistical analyses were performed using STATA version 19 for macOS (StataCorp, College Station, Texas 77845 USA).

## Results

During the study period, 12,144 neonates were born in HIS; 734 received antibiotics for suspected EONS, and 506 neonates met the inclusion criteria for likely EONS. Figure 1 shows the flow chart of patients included in the study. Among the 506 patients included, 304 were males (60.1%) and 202 females (39.9%), 2.6% were born preterm, and 2.8% had a low birth weight. Antibiotic treatment was initiated at birth in 29.4% of cases. Male neonates showed a higher incidence of jaundice compared to females (12% *vs.* 6.5%, *p* = 0.047). No neonates had positive blood cultures or experienced a fatal outcome (Table 1).

**Figure 1:**
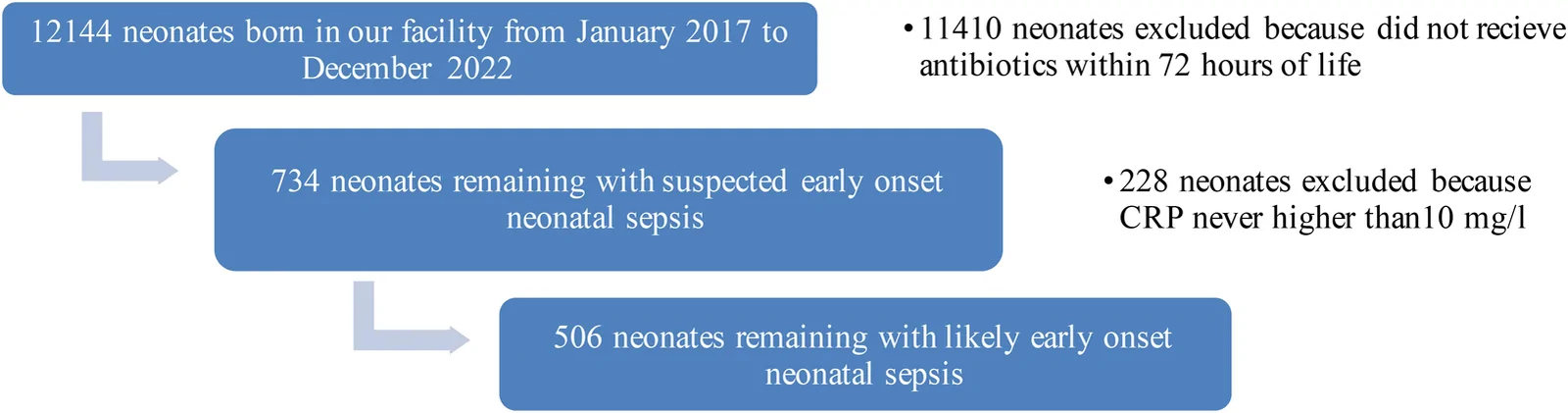
Flow chart of patient included in our study.

**Table 1:** General characteristics of neonates with CRP rise higher than 10 mg/L admitted to HIS neonatal unit (bruxelles, Belgium) from January 2017 to December 2022.

Neonatal characteristics	Males (*n* = 304)	Females (*n* = 202)	Total (*n* = 506)	Fisher's exact/ Mann–Whitney test *p*-value
Gestational age (n = 506)				
Late preterm (32–37 weeks)	10 (3.3%)	3 (1.5%)	13 (2.6%)	0.17
Normal term (>37 weeks)	294 (96.7%)	199 (98.5%)	493 (97.4%)	
Gestational age weeks (median, p25, p75)	40 (39, 41)	40 (39, 41)	40 (39, 41)	0.58
Birth weight (n = 506)				
Low birth weight (1,500–2,500 g)	11 (3.6%)	3 (1.5%)	14 (2.8%)	0.17
Normal birth weight (>2,500 g)	293 (96.4%)	199 (98.5%)	492 (97.2%)	
Birth weight Kg (median, p25, p75)	3.6 (3.23, 3.86)	3.43 (3.16, 3.73)	3.52 (3.2, 3.8)	0.006
Birth weight Z-score (median, p25, p75)	0.10 (−0.48, 0.79)	0.16 (−0.5, 0.81)	0.12 (−0.48, 0.79)	0.34
Clinical characteristics				
Respiratory distress (*n* = 499)	65 (21.7%)	44 (22.1%)	109 (21.8%)	0.91
Jaundice (*n* = 499)	36 (12.0%)	13 (6.5%)	49 (9.8%)	0.047
Fever (*n* = 503)	28 (9.3%)	19 (9.4%)	47 (9.3%)	1.0
Treatment started at birth (*n* = 506)	86 (28.3%)	63 (31.2%)	149 (29.4%)	0.49
Positive blood culture (*n* = 506)	0 (0%)	0 (0%)	0 (0%)	1.0
Contaminant positive blood culture (*n* = 506)	7 (2.3%)	4 (2.0%)	11 (2.2%)	
Delivery characteristics				
*Delivery type (n = 506)*				
Vaginal	141 (34.9%)	99 (49.0%)	240 (47.4%)	0.76
Vacuum-assisted	106 (46.4%)	70 (34.7%)	176 (34.8%)	
Caesarean	57 (18.7%)	33 (16.3%)	90 (17.8%)	
Infectious risk factors				
Maternal fever (*n* = 491)	56 (19.0%)	44 (22.3%)	100 (20.4%)	0.42
Maternal chorioamnionitis (*n* = 500)	8 (2.7%)	2 (1.0%)	10 (2.0%)	0.33
Prolonged membrane rupture (>18 h) (*n* = 490)	68 (23.0%)	38 (19.5%)	106 (21.6%)	0.37
Meconial amniotic liquid (*n* = 476)	113 (39.5%)	87 (45.8%)	200 (42.0%)	0.18
Maternal antibiotic treatment (*n* = 499)	154 (51.5%)	86 (43.0%)	240 (48.1%)	0.07
GBS colonisation (*n* = 482)	84 (29.2%)	42 (21.6%)	126 (26.1%)	0.07

At birth (T_0_), before antibiotic treatment initiation (Table 2), males and females had similar WBC with 15,581 WBC/µL (95% CI [14884, 16278]) for males and 16,328 WBC/µL (95% CI [15464, 17192]) for females. During the first 12 h of life, WBC counts increased in both sexes (471 cells/µL per hour in males and 512 cells/µL per hour in females, *p* = 0.001 for both), with no difference in the rate of increase between sexes. Between 12 and 24 h of life, WBC counts decreased in both groups (91 cells/µL per hour in males, *p* = 0.007, and 87 cells/µL per hour in females, *p* = 0.046) with no difference in the rate of decline between sexes. The left panel of Figure 2 illustrates these results.

**Table 2:** Piecewise mixed-effects linear spline regression model with random intercepts and slopes predicting WBC count and ANC in male and female neonates from birth to the initiation of antibiotics during the first 24 h of life. .

Fixed effects		WBC (cells/µL)				ANC (cells/µL)			
		Coefficient	*P*-value	[95% conf. interval]		Coefficient	*P*-value	[95% conf. interval]	
♂	Intercept (at birth)	15581	<0.001	14884	16278	7594	<0.001	7210	7978
♀		16328	<0.001	15464	17192	8387	<0.001	7900	8873
*Δ*♀-♂		747	0.187	−363	1857	793	0.012	173	1413
♂	H0-12 slope (/h)	471	<0.001	388	555	603	<0.001	536	671
♀		512	<0.001	407	616	673	<0.001	588	757
*Δ*♀-♂		40	0.555	−93	173	70	0.207	−38	178
♂	H12-24 slope (/h)	−91	0.007	−157	−25	−142	<0.001	−202	−82
♀		−87	0.046	−173	−2	−166	<0.001	−243	−88
*Δ*♀-♂		3	0.952	−105	111	−24	0.631	−122	74
Random effects									
Variance of intercept		2.16*10^7^		.	.	3.8*10^6^		1.06*10^6^	1.35*10^7^
Variance of slopes									
H0-12		1.72*10^5^		.	.	96680		46446	2.01*10^5^
H12-24		2.62*10^5^		.	.	26659		2080	3.42*10^5^
Conditional intraclass correlation		0.69		0.69	0.69	0.46		0.08	0.89
Conditional R^2^		0.77				0.84			
Marginal R^2^		0.18				0.42			
Number of patients		474 913				437 757			
Number of observations									

♂: male neonates; ♀: female neonates Delta ♀-♂: difference between male and female neonates. Slopes are expressed in cells/microlitre/hour. Intercepts are expressed in cells/microlitre.

**Figure 2:**
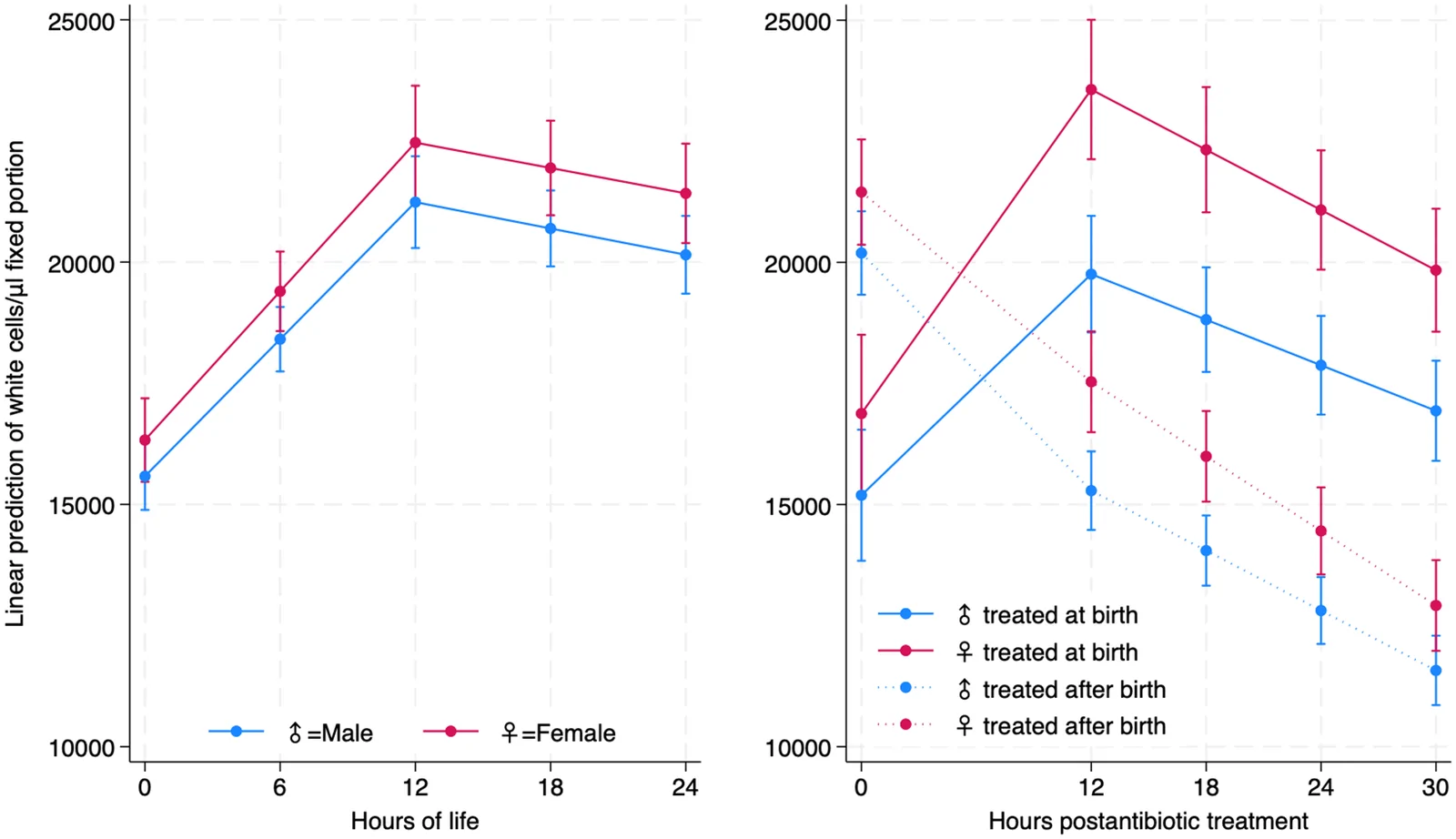
Graphic representation of WBC count according to regression models as seen in Tables 2 (left part) and 3 (right part). **On the left part**: Piecewise mixed linear spline regression model with random effects on intercept and slopes predicting white blood cells (/µL) in male and female neonates from birth to antibiotic start in the first 24 h of life (474 patients, 913 observations). **On the right part**: Piecewise mixed linear spline regression model with random effects on intercept and slopes predicting white blood cells (/µL) in male and female neonates from antibiotic start to 30 h thereafter (498 patients, 1,130 observations).

Female neonates had higher neutrophil counts at birth (T_0_) compared to males, with a mean difference of 793 cells/µL (*p* = 0.012). During the first 12 h of life, ANC increased in both sexes (603 cells/µL per hour in males and 673 cells/µL per hour in females, *p* = 0.001 for both), with no difference in the rate of increase between sexes. From 12 to 24 h, ANC decreased in both groups (142 cells/µL per hour in males and 166 cells/µL per hour in females, *p* = 0.001 for both), again showing no difference in the rate of decline. The left panel of Figure 3 illustrates these results.

**Figure 3:**
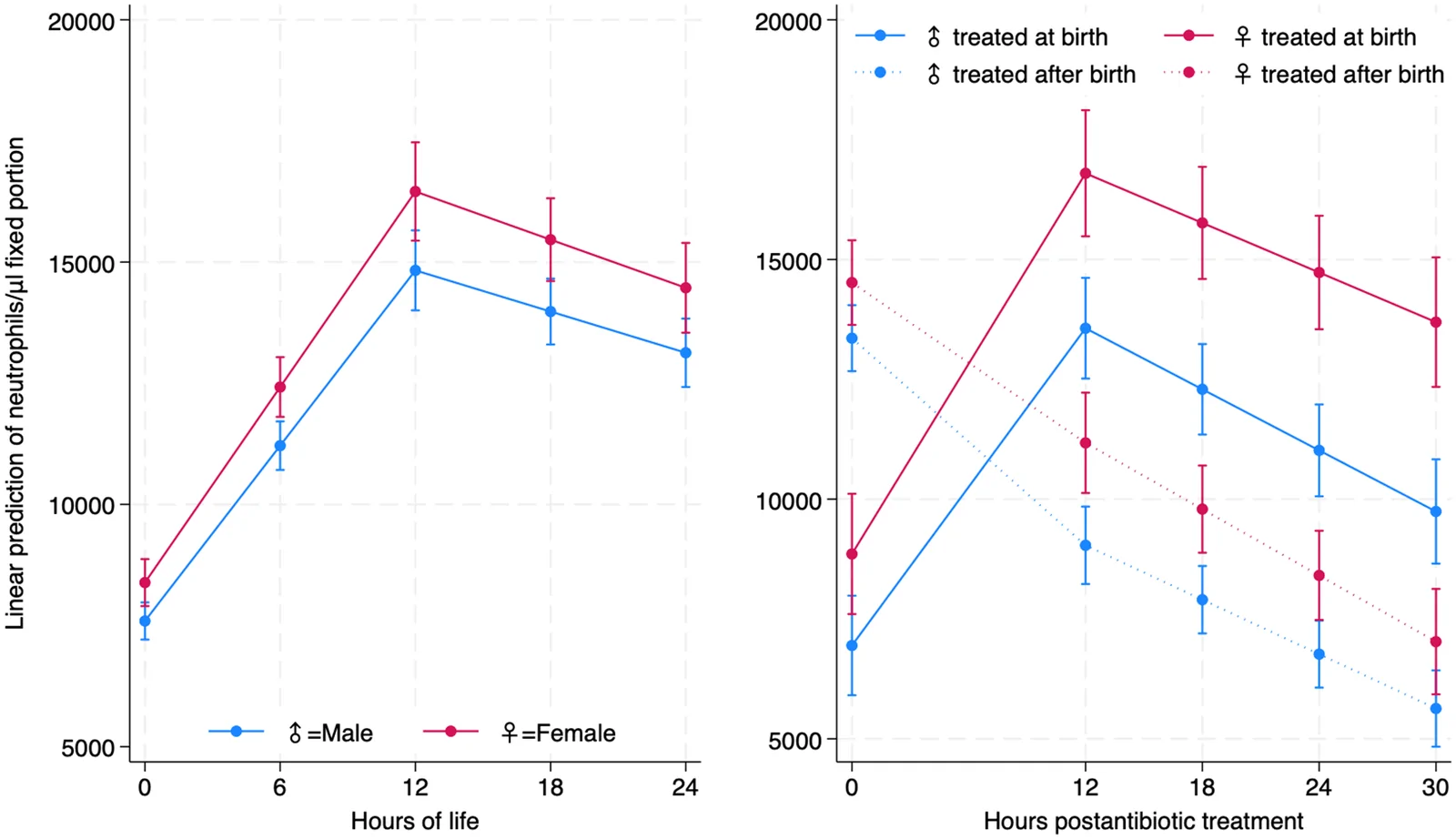
Graphic representation of ANC according to regression models as seen in Tables 2 (left part) and 3 (right part). **On the left part**: Piecewise mixed linear spline regression model with random effects on intercept and slopes predicting neutrophils (cells/µL) in male and female neonates from birth to antibiotic start in the first 24 h of life (437 patients, 757 observations). **On the right part**: Piecewise mixed linear spline regression model with random effects on intercept and slopes predicting neutrophils (cells/µL) in male and female neonates from antibiotic start to 30 h thereafter (449 patients, 778 observations).

Among neonates who received antibiotic treatment at birth (T_0_) (Table 3), mean WBC count was 15,191 cells/µL (95% CI [13838, 16545]) for males *vs.* 16,879 cells/µL (95% CI [15253, 18505]) for females, with no difference between sexes (*p* = 0.118). During the first 12 h of treatment, WBC count increased in both groups (380 cells/µL per hour in males and 557 cells/µL per hour in females, *p* = 0.001 for both), with a faster increase observed in females (177 cells/µL per hour, *p* = 0.020). Between 12 and 30 h after treatment initiation, WBC counts decreased in both sexes (157 cells/µL per hour in males and 207 cells/µL per hour in females, *p* = 0.001 for both), with no difference in the rate of decline between sexes.

**Table 3:** Piecewise mixed-effects linear spline regression model with random intercepts and slopes predicting WBC count and ANC in male and female neonates, based on the timing of antibiotic initiation (at birth or after birth), and from antibiotic initiation to 30 h thereafter.

Fixed effects		WBC (cells/µL)				ANC (cells/µL)			
		Coefficient	*P*-value	[95% conf. interval]		Coefficient	*P*-value	[95% conf. interval]	
Antibiotics started at birth									
♂	Intercept (at antibiotic start)	15191	<0.001	13838	16545	6948	<0.001	5910	7985
♀		16879	<0.001	15253	18505	8857	<0.001	7603	10112
*Δ*♀-♂		1688	0.118	−428	3803	1910	0.022	282	3537
♂	H0-12 slope (/h)	380	<0.001	285	475	552	<0.001	468	635
♀		557	<0.001	443	672	662	<0.001	555	769
*Δ*♀-♂		177	0.020	28	326	110	0.111	−26	246
♂	H12-30 slope (/h)	−157	<0.001	−203	−110	−212	<0.001	−270	−155
♀		−207	<0.001	−266	−149	−172	<0.001	−246	−99
*Δ*♀-♂		−51	0.185	−125	24	40	0.403	−54	134
Antibiotics started after birth									
♂	Intercept (at antibiotic start)	20193	<0.001	19331	21055	13359	<0.001	12670	14048
♀		21451	<0.001	20364	22538	14519	<0.001	13636	15401
*Δ*♀-♂		1258	0.076	−129	2646	1160	0.042	40	2279
♂	H0-12 slope (/h)	−409	<0.001	−474	−344	−360	<0.001	−427	−293
♀		−326	<0.001	−411	−242	−279	<0.001	−368	−190
*Δ*♀-♂		82	0.131	−25	189	81	0.153	−30	193
♂	H12-30 slope (/h)	−206	<0.001	−240	−172	−189	<0.001	−235	−143
♀		−257	<0.001	−300	−213	−230	<0.001	−296	−165
*Δ*♀-♂		−50	0.076	−106	5	−41	0.311	−121	38
Random effects									
Variance of intercept		3.5*10^7^		.	.	1.77*10^7^		1.34*10^7^	2.33*10^7^
Variance of slopes									
H0-12		1.2*10^5^		.	.	72499		33381	1.57*10^5^
H12-30		9610		.	.	11963		1434	99763
Conditional intraclass correlation		0.89		0.89	0.89	0.86		0.57	0.96
Conditional R^2^		0.90				0.90			
Marginal R^2^		0.26				0.35			
Number of patients		498				449			
Number of observations		1130				778			

♂: male neonates; ♀: female neonates Delta ♀-♂: difference between male and female neonates. Slopes are expressed in cells/microlitre/hour. Intercepts are expressed in cells/microlitre.

For neonates who received antibiotic treatment after birth, mean WBC count at antibiotic initiation (T_0_) was higher compared to those who received treatment at birth (20,193 cells/µL; 95% CI [19331, 21055] for males vs. 21,451 cells/µL; 95% CI [20364, 22538] for females). During the first 12 h of antibiotic treatment, WBC counts decreased in both sexes (409 cells/µL per hour in males and 326 cells/µL per hour in females, *p* = 0.001 for both), in contrast to the increase observed in neonates treated at birth. No difference in the rate of decline was found between the sexes during this period. Between 12 and 30 h after treatment initiation, WBC count continued to decrease in both groups (206 cells/µL per hour in males and 257 cells/µL per hour in females, *p* = 0.001 for both), with no difference in the rate of decline between sexes. The right panel of Figure 2 illustrates these results.

In neonates who received antibiotic treatment at birth, mean ANC at T_0_ (birth and antibiotic initiation) was 6,948 cells/µL (95% CI [5910, 7985]) for males vs. 8,857 cells/µL (95% CI [7603, 10112]) for females (*p* = 0.022). During the first 12 h of antibiotic treatment, ANC increased in both sexes (552 cells/µL per hour in males and 662 cells/µL per hour in females, *p* = 0.001 for both), with no difference in the rate of increase between sexes. Between 12 and 30 h after treatment initiation, ANC decreased in both groups (212 cells/µL per hour in males and 172 cells/µL per hour in females, *p* = 0.001 for both), with no difference in the rate of decline between sexes.

In neonates who received antibiotic treatment after birth, mean ANC at antibiotic initiation (T_0_) was higher compared to those who started treatment at birth (13,359 cells/µL; 95% CI [12670, 14048] for males and 14,519 cells/µL; 95% CI [13636, 15401] for females). Female neonates also had higher ANC at the start of treatment than males, with a difference of 1,160 cells/µL (*p* = 0.042). During the first 12 h of antibiotic treatment, ANC decreased in both sexes (360 cells/µL per hour in males and 279 cells/µL per hour in females, *p* = 0.001 for both), which contrasted with the increase observed in neonates treated at birth. No difference in the rate of decrease was observed between the sexes during this period. Between 12 and 30 h after treatment initiation, ANC continued to decrease in both groups (189 cells/µL per hour in males and 230 cells/µL per hour in females, *p* = 0.001 for both), with no difference in the rate of decline between sexes. The right panel of Figure 3 illustrates these results.

## Discussion

In this study, we observed that female neonates consistently had higher ANC counts, and, to a lesser extent, WBC counts, than to their male counterparts, whether at birth, before any antibiotic initiation, or after initiation. Schmutz et al. demonstrated a similar difference in a substantial general neonatal population comprising an 18-hospital healthcare system in the United States in 2008, particularly for ANC (7). Interestingly, we also found a sex difference in the kinetics of WBC and ANC, with females showing a more pronounced elevation in WBC during the first 12 h of antibiotic treatment when treatment was initiated at birth, but not when initiated afterward.

While data in neonates are limited, sensitive hormonal assays in infancy and childhood consistently detect estradiol concentrations that are higher in girls than in boys, even before puberty (15). In cord blood, maternal/placental estrogens (especially estriol) are high and show higher levels in girls (16), but the extent to which residual maternal estrogens influence neonatal ANC — independent of sex — remains unclear. A possible explanation is that female neonates have a higher baseline ANC and mount a transiently greater rise in WBC after antibiotic initiation. However, direct evidence linking sex-specific neonatal estrogen levels to ANC kinetics is lacking and warrants further studies involving neonatal hormone measurements, receptor expression assays, and mechanistic *in vitro* models.

Rather than sex hormones acting alone, genetics has become increasingly important to explain such inflammatory sex disparities. In particular, specific pattern recognition receptors, such as Toll-like receptors (TLRs), which are encoded on the X chromosome, have been shown to cause sex-specific immune responses (17, 18). Sex differences in TLR responsiveness have already been documented in neonates, suggesting that dimorphic innate immune activation is present early in life (19). Interleukin (IL)-8, which is produced in response to the activation of the TLR pathway, was found to be higher in females than in males after whole blood stimulation with lipopolysaccharide (20). Given that IL-8 is the main neutrophil chemoattractant, a higher level of IL-8 may explain the higher ANC observed in female neonates in response to sepsis. In umbilical cord blood samples from term neonates, IRAK1 gene and protein expression were significantly higher in females than in males, with increased cytosolic IRAK1 expression. This increased expression of IRAK1 could also be responsible, for the sex-specific responses to infection observed in neonates (21). Further studies, including measurements of plasma levels of TLR pathway cytokines in neonates with confirmed EONS are warranted to test this hypothesis.

Consistent with previously mentioned literature, we observed that WBC count and ANC followed a predictable pattern in untreated neonates at birth, peaking within the first 12 h of life and declining thereafter (5–9). This physiological leukocytosis is a regular part of the stress response during delivery and the transition to extrauterine life. The initial rise in WBC and ANC has been linked to demargination and increased bone marrow output triggered by cortisol and cytokine release due to labor (4, 7, 8). Interestingly, neonates who received antibiotics at birth also exhibited an increase in WBC count and ANC during the first 12 h of life. These data suggest that the physiological postnatal rise in WBC and ANC is not influenced by early initiation of antibiotic treatment. Consequently, WBC count and ANC during this period may have limited diagnostic information in likely EONS, aligning with observations reported in previous studies (22, 23).

An additional finding was that for every two female neonates, three male neonates were included, suggesting a higher proportion of male neonates receiving treatment for EONS. A similar trend was reported in a retrospective cross-sectional study of 757,979 late-preterm and full-term neonates from 2014 to 2018 across Europe, the US, and Australia showing similar sex imbalance among proven and not proven EONS (24). The higher proportion of male neonates treated for suspected EONS may reflect both biological and clinical factors. Male neonates may be more susceptible to infectious complications because of sex-specific differences in innate immune function. In particular, differences in X-linked immune gene dosage and Toll-like receptor signaling may contribute to a less pronounced inflammatory response in males. With females exhibiting higher IL-8 responses and higher IRAK-1 expression following innate immune stimulation (20, 21). However, our findings cannot distinguish between increased biological susceptibility to infection and differences in diagnostic or therapeutic thresholds. Giannoni et al. reported an overall incidence of confirmed EONS of 0.49 cases per 1,000 live births (i.e., 375/21,703 = 1.7% versus 0%) (24). The absence of confirmed EONS cases in our cohort might be explained by the fact that our study was conducted at a secondary hospital, where critically ill patients, including neonates and women with high-risk pregnancies, are typically transferred to tertiary care hospitals.

Our findings also have several potential clinical implications. First, WBC and ANC should be interpreted according to postnatal age because both parameters undergo a marked physiological increase during the first 12 h of life, which was also observed in neonates receiving antibiotics at birth. This limits their diagnostic value when measured during this early physiological period. Second, the consistently higher ANC observed in female neonates suggests that sex may be another factor to consider when interpreting neonatal inflammatory markers. However, our findings do not support the introduction of sex-specific diagnostic thresholds at this stage. Prospective multicenter studies including microbiologically confirmed EONS are warranted to validate these sex-specific trajectories and determine whether they can improve diagnostic accuracy or reduce unnecessary antibiotic exposure.

### Strengths and limitations

Several limitations should be considered when interpreting our findings. First, this was a retrospective, monocentric study, which limits the generalizability of our results. Second, our cohort consisted of neonates with likely EONS rather than microbiologically confirmed infection. Although blood cultures were systematically obtained whenever technically feasible, no culture-positive EONS case was identified. Therefore, we cannot exclude misclassification of non-infectious inflammatory conditions as likely EONS, and our findings should not be extrapolated to microbiologically confirmed EONS. Third, our hospital is a secondary-care center, and very preterm neonates and neonates with very low birth weight are generally transferred to tertiary centers. Consequently, our cohort predominantly represents term and late-preterm neonates requiring evaluation and treatment for suspected EONS. Fourth, antibiotic initiation was determined by the treating clinicians rather than randomly assigned. The timing of treatment may therefore be influenced by disease severity, clinical presentation, or other unmeasured factors, limiting any causal interpretation of the differences observed between neonates treated at birth and those treated later. Fifth, the retrospective design limited our ability to account for all factors known to influence neonatal WBC and ANC, including the precise volume obtained for blood culture and other potentially unmeasured maternal, perinatal, and neonatal factors. Finally, the number and timing of blood samples were determined by clinical care, which may have introduced variability in the longitudinal measurements. Despite these limitations, the study provides longitudinal data on WBC and ANC kinetics in a relatively large cohort of neonates with likely EONS and provides, to our knowledge, the first description of these trajectories following initiation of antibiotic therapy.

In conclusion, female neonates with likely EONS had higher ANC values than male neonates and showed a more pronounced increase in WBCs during the first 12 h after antibiotic initiation when treatment was started at birth. These findings suggest that sex may influence early inflammatory cell trajectories in neonates and should be considered when interpreting WBC and ANC in the context of suspected EONS.

## Data Availability

The raw data supporting the conclusions of this article will be made available by the authors, under reasonable request.
